# Supplementary material for: petBrain: a new pipeline for amyloid, Tau tangles and neurodegeneration quantification using PET and MRI
Source: Alzheimers Res Ther. 2025 Sep 30;17:209. doi: 10.1186/s13195-025-01839-y (PMC12482854; doi:10.1186/s13195-025-01839-y)
Supplement: Supplementary file 1 — Supplementary Material 1 [file 13195_2025_1839_MOESM1_ESM.docx]

petBrain: A New Pipeline for Amyloid, Tau Tangles and Neurodegeneration Quantification Using PET and MRI

# *supplementary material*

*Pierrick Coupé^1^, Boris Mansencal^1^, Floréal Morandat^1^, Sergio Morell-Ortega^2^, Nicolas Villain^3,4^, Jose V. Manjón^2^, Vincent Planche^5,6^*

*1 CNRS, Univ. Bordeaux, Bordeaux INP, LABRI, UMR5800, F-33400 Talence, France*

*2 ITACA, Universitat Politècnica de València, 46022 Valencia, Spain*

*3 AP-HP Sorbonne Université, Hôpital Pitié-Salpêtrière, Department of Neurology, Institute of Memory and Alzheimer’s Disease, Paris, France*

*4 Institut du Cerveau - ICM, Sorbonne Université, INSERM U1127, CNRS 7225, Paris, France
5 CHU Bordeaux, Service de Neurologie des Maladies Neurodégénératives, Centre Mémoire Ressources Recherche, F-33000 Bordeaux, France*

*6 Univ. de Bordeaux, CNRS, UMR 5293, Institut des Maladies Neurodégénératives, F-33000 Bordeaux, France*

# GAAIN Datasets description

In this section, we provide additional information on the GAAIN data used in our study.

- **PiB dataset (Klunk et al. 2015)**: This dataset consists of 79 paired T1-w MRI and PiB PET scans acquired 50–70 minutes post-injection. It includes data from 34 young cognitively normal (yCN) controls and 45 patients with AD. The 34 yCN were under the age of 45 (mean age: 31 ± 6 years; range: 22–43) and were deemed cognitively normal based on a standard neuropsychological and clinical evaluation. The 45 AD patients were diagnosed according to the 1984 NINCDS-ADRDA criteria, had a Clinical Dementia Rating (CDR) Global Score of 0.5 or 1 and were amyloid-positive. Their amyloid status was based on PiB-positive cutoff method. Their mean age was 67 ± 10 years (range: 50–89).
- **FBP** **Dataset (Navitsky et al. 2018)**: This dataset consists of 46 paired T1-w MRI scans and PiB PET (50–70 minutes post-injection) and ¹⁸F-Florbetapir PET (50–60 minutes post-injection) scans from 13 yCN and 33 elderly CN. The 13 yCN were under the age of 35 (mean age: 27 ± 4 years; range: 21–35) and had a Mini-Mental State Examination (MMSE) score ranging from 29 to 30. The 33 elderly subjects were further categorized as follows: 17 AD patients (mean age: 67.1 ± 7.1 years; range: 51–76) with MMSE range: 8–26, 7 individuals with MCI (mean age: 80 ± 8 years; range: 64–89) with MMSE range: 25–28, 3 at-risk elderly subjects (mean age: 80 ± 3 years; range: 78–83) with MMSE range: 28–30 and 6 elderly controls (mean age: 63 ± 8 years; range: 51–75) with MMSE range: 27–30.
- **FTM Dataset (Battle et al. 2018):** This dataset consists of 74 paired T1-w MRI scans and PiB PET (50–70 minutes post-injection) and ¹⁸F-Flutemetamol PET (90–110 minutes post-injection) scans from 24 yCN and 50 elderly subjects. The 24 yCN were under the age of 45 (mean age: 37 ± 5 years; range: 30–45). The 50 elderly subjects were categorized as follows: 20 patients with AD dementia (mean age: 69 ± 10 years; range: 60–82) 20 individuals with amnestic MCI (mean age: 73 ± 7 years; range : 57–83) and 10 older healthy controls (mean age: 57 ± 11 years; range : 47–75).
- **FBB Dataset (Rowe et al. 2017):** This dataset consists of 35 paired T1-w MRI scans and ¹⁸F-Florbetaben PET (90–110 minutes post-injection) and PiB PET (50–70 minutes post-injection) scans from 25 elderly subjects and 10 yCN. The 10 yCN were under the age of 45 (mean age: 33 ± 8 years) and had an MMSE score higher than 28. The 25 elderly subjects were categorized as follows: 6 healthy elderly controls (mean age: 71 ± 8 years, MMSE: 29 ± 1), 9 individuals with MCI (mean age: 72 ± 5 years, MMSE: 28 ± 2), 8 patients with AD (mean age: 69 ± 6 years, MMSE: 23 ± 3) and 2 patients with frontotemporal dementia (FTD) (mean age: 74 ± 8 years, MMSE: 23 ± 1).
- **NAV Dataset (Rowe et al. 2016):** This dataset consists of 55 paired T1-weighted MRI scans and PiB PET (50–70 minutes post-injection) and ¹⁸F-NAV4694 PET scans from 10 yCN and 45 CN. The 10 yCN were under the age of 45 (mean age: 33 ± 8 years) and had an MMSE score higher than 28. The 45 elderly subjects were categorized as follows: 25 elderly CN (mean age: 74 ± 8 years, MMSE: 29 ± 1), 10 individuals with MCI (mean age: 75 ± 9 years, MMSE: 27 ± 3), 7 AD patients (mean age: 73 ± 11 years, MMSE: 24 ± 2) and 3 FTD patients (mean age: 68 ± 5 years, MMSE: 27 ± 1).
- **FTP Dataset (Villemagne et al. 2023)**: This dataset contains 100 paired T1-w MRI scans and ¹⁸F-Flortaucipir tau PET images from 50 CN controls and 50 patients with AD. The 50 AD patients were aged 65 and older and had a clinical diagnosis of probable AD with an amnestic phenotype. They also had a positive PiB amyloid PET scan and no additional neurological diagnoses. The 50 CN A- were age- and sex-matched to the AD patient group and had a negative amyloid PET scan.

# Centiloid subject-specific mask

To establish the list of structures to include into the petBrain Centiloid mask, we estimated Cohen’s d scores for each structure between the yCN A- and Dementia A+ of the PiB dataset. First, we selected all the structures with a Cohen’s d score > 5. Afterwards, we added missing contralateral structures to obtain a symmetrical mask. The final list of selected structures is presented in Supplementary Table 1.

Supplementary Table 1: Cohen’s d scores for each structure on the young yCN A- and Dementia A+ of the PiB dataset.

| Structure Name | Cohen’s d | Label number |
| --- | --- | --- |
| \| Right anterior cingulate gyrus \| \| --- \| \| Left anterior cingulate gyrus \| \| Left medial frontal cortex \| \| Left precuneus \| \| Right medial frontal cortex \| \| Right sup. frontal gyrus medial segment \| \| Right Accumbens \| \| Right middle cingulate gyrus \| \| Right sup. temporal gyrus \| \| Right middle temporal gyrus \| \| Left subcallosal area \| \| Left sup. frontal gyrus medial segment \| \| Right precuneus \| \| Right posterior cingulate gyrus \| \| Right middle frontal gyrus \| \| Left posterior cingulate gyrus \| \| Left angular gyrus \| \| Right subcallosal area \| \| Left middle cingulate gyrus \| \| Right anterior insula \| \| Right fusiform gyrus \| \| Right inf. temporal gyrus \| \| Left middle temporal gyrus \| \| Left supramarginal gyrus \| \| Right frontal operculum \| \| Left anterior insula \| \| Right supramarginal gyrus \| \| Right triangular inf. frontal gyrus \| \| Right angular gyrus \| \| Left sup. temporal gyrus \| \| Left frontal operculum \| \| Left Accumbens \| \| Left inf. temporal gyrus \| \| Left middle frontal gyrus \| \| Left triangular inf. frontal gyrus \| \| Left fusiform gyrus \| | \| 7.33 \| \| --- \| \| 6.81 \| \| 6.58 \| \| 6.40 \| \| 6.33 \| \| 6.27 \| \| 6.06 \| \| 5.94 \| \| 5.85 \| \| 5.78 \| \| 5.75 \| \| 5.67 \| \| 5.66 \| \| 5.65 \| \| 5.63 \| \| 5.58 \| \| 5.57 \| \| 5.56 \| \| 5.48 \| \| 5.41 \| \| 5.40 \| \| 5.33 \| \| 5.32 \| \| 5.31 \| \| 5.23 \| \| 5.20 \| \| 5.19 \| \| 5.11 \| \| 5.02 \| \| 4.91 \| \| 4.90 \| \| 4.88 \| \| 4.82 \| \| 4.80 \| \| 4.72 \| \| 4.71 \| | \| 100 \| \| --- \| \| 101 \| \| 141 \| \| 169 \| \| 140 \| \| 152 \| \| 23 \| \| 138 \| \| 200 \| \| 154 \| \| 187 \| \| 153 \| \| 168 \| \| 166 \| \| 142 \| \| 167 \| \| 107 \| \| 186 \| \| 139 \| \| 102 \| \| 122 \| \| 132 \| \| 155 \| \| 195 \| \| 118 \| \| 103 \| \| 194 \| \| 204 \| \| 106 \| \| 201 \| \| 119 \| \| 30 \| \| 133 \| \| 143 \| \| 205 \| \| 123 \| |

1. PiB calibration

|  |
| --- |
| Supplementary Figure 1: **petBrain validation for Centiloid (CL) measure: calibration with the PiB tracer.** The used PiB dataset was composed of 34 young A- cognitively normal subject and 45 A+ patients with dementia. |

1. Other Amyloid tracers’ calibration

|  |  |
| --- | --- |
|  |  |
|  |  |
|  |  |

Supplementary Figure 2: Calibration of the FBP, FBB, FTM and NAV amyloid tracers using the corresponding Centiloid Project datasets.

# Centiloid subject-specific mask

For the CenTauR mask, we used the following list of structures – entorhinal area, amygdala, parahippocampal gyrus, fusiform gyrus, inferior and middle temporal gyrus, and temporal pole.

Supplementary Table 2: Cohen’s d scores for each structure on the young CN A- and old AD A+ of the FTP dataset.

| Structure Name | Cohen’s D | Label number |
| --- | --- | --- |
| \| Left parahippocampal gyrus \| \| --- \| \| Left entorhinal area \| \| Left Amygdala \| \| Right entorhinal area \| \| Right parahippocampal gyrus \| \| Right Amygdala \| \| Right inf. temporal gyrus \| \| Left inf. temporal gyrus \| \| Right fusiform gyrus \| \| Left middle temporal gyrus \| \| Left fusiform gyrus \| \| Right middle temporal gyrus \| \| Left temporal pole \| \| Right temporal pole \| | \| 2.94 \| \| --- \| \| 2.91 \| \| 2.65 \| \| 2.63 \| \| 2.59 \| \| 2.54 \| \| 2.28 \| \| 2.18 \| \| 2.08 \| \| 2.02 \| \| 2.01 \| \| 1.99 \| \| 1.95 \| \| 1.86 \| | \| 171 \| \| --- \| \| 117 \| \| 32 \| \| 116 \| \| 170 \| \| 31 \| \| 132 \| \| 133 \| \| 122 \| \| 155 \| \| 123 \| \| 154 \| \| 203 \| \| 202 \| |

Supplementary Table 3: Comparison of the predefined CenTauR masks with our petBrain subject-specific mask on the FTP dataset.

| Mask | R^2^ |
| --- | --- |
| Universal | 0.91 |
| Mesial-temporal | 0.90 |
| Meta-temporal | 0.98 |
| Temporo-parietal | 0.91 |
| Frontal | 0.68 |

# CenTauR FTP calibration

|  |
| --- |
| Supplementary Figure 3: **petBrain validation for CenTauRz (CTRz) measure: calibration with FTP tracer**. The used FTP dataset was composed of 50 young A- cognitively normal subject and 50 old A+ patients with AD dementia. |

# Validation of PET measurements without Partial Volume Correction (PVC)

In this section, we evaluated the influence of the partial volume correction (PVC) step on the outcomes of our processing pipeline. As illustrated in Supplementary Figure 4, the correlation between petBrain and B-PIP remained highly consistent whether PVC was applied (see Figure 3) or not. Similarly, analyses involving Centiloid and CenTauR metrics in relation to AD fluid biomarkers yielded comparable results with and without PVC (Supplementary Table 4). This observation also extended to cognitive scores, for which the associations remained largely unchanged across conditions (Supplementary Table 5). Collectively, these complementary analyses demonstrate a strong concordance between results obtained with and without PVC, supporting the robustness and reliability of the proposed PVC methodology. Although the differences observed were minimal, we noted a consistent trend favoring the application of PVC.

The small variations in Centiloid and CentaurZ values can be attributed to the averaging over large anatomical masks — such as the CenTauR mask, encompassing over 50,000 voxels — which inherently reduces the local impact of partial volume effects. Consequently, quantifications remain largely stable across conditions.

Despite the marginal impact on numerical outcomes, we elected to retain the PVC step in our pipeline due to its contribution to improved image clarity. This enhancement is particularly relevant for the visual assessment of amyloid or tau deposition, providing added value in both clinical and research contexts (see Supplementary Figure 7 for an illustrative example).

|  |  |
| --- | --- |
|  |  |
| Supplementary Figure 4: Comparison of Centiloid values and Tau SUVr obtained with petBrain (without PVC step) and the value provided by B-PIP. | |

We repeated the same comparison using the SPM-based pipelines instead of B-PIP. The results are shown in Supplementary Figure 5, and similar conclusions apply. Furthermore, we assessed the correlation between petBrain results with and without PVC. As shown in Supplementary Figure 6, the results remain highly consistent, with R² = 1 and ICC = 1, confirming the robustness of petBrain to PVC application.

|  |  |
| --- | --- |
|  |  |
| Supplementary Figure 5: Comparison of Centiloid and CenTauR values obtained with petBrain (without PVC step) and the values obtained with SPM-based pipelines. | |

|  |  |
| --- | --- |
|  |  |
| Supplementary Figure 6: Comparison of Centiloid and CenTauR values obtained with petBrain with and without PVC step. | |

Supplementary Table 4: **Association of Centiloid (CL), tau SUVr, CenTauRz (CTRz) produced by petBrain (with and without PVC step) with AD fluid biomarkers.** The linear mixed models were adjusted for age, sex, and APOEε4. The used metrics were p-value of the F-test and R^2^.

|  | p-value | R^2^ |  |
| --- | --- | --- | --- |
| CL ~ CSF Aβ42/40 (N=353) | | |  |
| petBrain | 2.46e-58 | 0.546 |  |
| petBrain wihtout pvc | 2.59e-58 | 0.545 |  |
| CL ~ CSF p-tau (N=350) | | |  |
| petBrain | 7.1e-27 | 0.311 |  |
| petBrain wihtout pvc | 9.9e-27 | 0.307 |  |
| CL ~ log(Plasma p-t217) (N=397) | | |  |
| petBrain | 2.49e-65 | 0.542 |  |
| petBrain wihtout pvc | 8.28e-64 | 0.534 |  |
| Meta-temporal tau SUVr ~ log(Plasma p-t217) (N=397) | | | |
| petBrain | | 8.23e-54 | 0.475 |
| petBrain wihtout pvc | | 1.03e-53 | 0.475 |
| CTRz ~ log(Plasma pT217) (N=397) | | | |
| petBrain | | 1.48e-56 | 0.492 |
| petBrain wihtout pvc | | 1.61e-56 | 0.490 |

Supplementary Table 5: **Associations between cognitive scores (CDR-sb, MMSE, MoCA) and petBrain without and with pvc.** Models were adjusted for age, sex, APOEε4 status, and education.

|  | p-value | R^2^ |
| --- | --- | --- |
|  | CDR-sb (N = 718) | |
|  | wihtout pvc / with pvc | wihtout pvc / with pvc |
| A (CL) | 1.52e-26 / 9.47e-27 | 0.168 / 0.169 |
| T_2_ (CTRz) | 6.7e-43 / 3.16e-43 | 0.253 / 0.254 |
| N (HAVAs) | 3.65e-55 | 0.310 |
| A/T_2_/N | **7.67e-64 / 6.33e-64** | **0.356^a,b^ / 0.356^a,b^** |
|  | MMSE (N = 719) | |
| A (CL) | 3.54e-30 / 1.13e-30 | 0.185 / 0.190 |
| T_2_ (CTRz) | 3.81e-45 / 1.52e-45 | 0.264 / 0.265 |
| N (HAVAs) | 4.03e-45 | 0.263 |
| A/T_2_/N | **2.81e-57 / 2.01e-57** | **0.328^a^ / 0.328^a^** |
|  | MoCA (N = 647) | |
| A (CL) | 2.66e-34 / 1.56e-34 | 0.230 / 0.231 |
| T_2_ (CTRz) | 6.37e-49 / 1.31e-49 | 0.310 / 0.311 |
| N (HAVAs) | 8.62e-51 | 0.316 |
| A/T_2_/N | **8e-61 / 6.69e-61** | **0.373^a^ / 0.373^a^** |

^a^ Indicates a significant difference (adjusted p-value<0.0125) with model A using a Steiger’s test on R^2^

^b^ Indicates a significant difference (adjusted p-value< 0.0125) with model T_2_ using a Steiger’s test on R^2^

The Steiger’s test has been corrected for multiple comparison using Bonferroni correction

| *Without PVC* | *With PVC* |
| --- | --- |
| *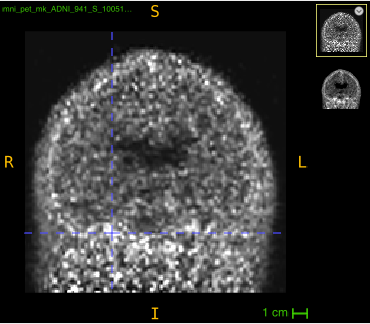* | *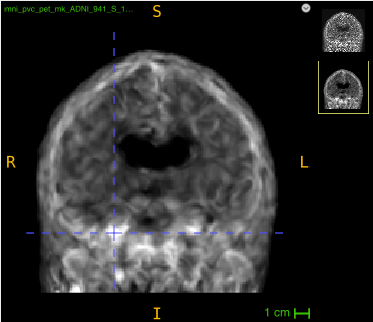* |
| *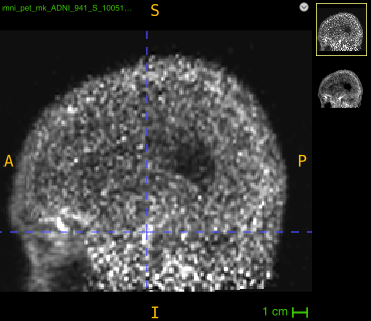* | *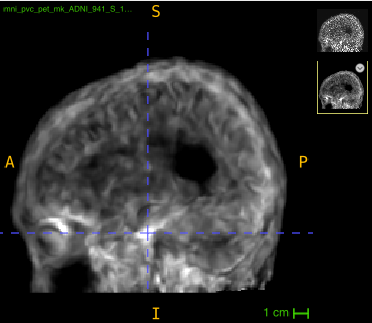* |

Supplementary Figure 7. Example of Tau PET imaging (MK tracer) in an amyloid-positive subject from the ADNI cohort presenting with clinical symptoms of Alzheimer’s disease. The application of PVC enhances the visualization of Tau deposition, particularly in the parahippocampal gyrus, allowing for improved anatomical visualization of tracer uptake*.*

# Automatically generated report by the web-based VolBrain platform

|  |
| --- |
| Supplementary Figure 8: Example of PDF report produced by petBrain about an amyloid-positive patient with dementia in ADNI |
